# A common goal to CARE: Cancer Advocates, Researchers, and Clinicians Explore current treatments and clinical trials for breast cancer brain metastases

**DOI:** 10.1038/s41523-021-00326-5

**Published:** 2021-09-14

**Authors:** Natalie S. Joe, Christine Hodgdon, Lianne Kraemer, Kristin J. Redmond, Vered Stearns, Daniele M. Gilkes

**Affiliations:** 1 Department of Oncology, The Sidney Kimmel Comprehensive Cancer Center, The Johns Hopkins University School of Medicine, Baltimore, MD USA; 2 Cellular and Molecular Medicine Program, The Johns Hopkins University School of Medicine, Baltimore, MD USA; 3 INSPIRE (Influencing Science through Patient-Informed Research & Education) Advocacy Program, The Johns Hopkins University School of Medicine, Baltimore, MD USA; 4 Department of Radiation Oncology and Molecular Radiation Sciences, The Johns Hopkins University School of Medicine, Baltimore, MD USA; 5 Department of Chemical and Biomolecular Engineering, The Johns Hopkins University, Baltimore, MD USA

**Keywords:** Metastasis, Breast cancer, Targeted therapies, Radiotherapy

## Abstract

Breast cancer is the most commonly diagnosed cancer in women worldwide. Approximately one-tenth of all patients with advanced breast cancer develop brain metastases resulting in an overall survival rate of fewer than 2 years. The challenges lie in developing new approaches to treat, monitor, and prevent breast cancer brain metastasis (BCBM). This review will provide an overview of BCBM from the integrated perspective of clinicians, researchers, and patient advocates. We will summarize the current management of BCBM, including diagnosis, treatment, and monitoring. We will highlight ongoing translational research for BCBM, including clinical trials and improved detection methods that can become the mainstay for BCBM treatment if they demonstrate efficacy. We will discuss preclinical BCBM research that focuses on the intrinsic properties of breast cancer cells and the influence of the brain microenvironment. Finally, we will spotlight emerging studies and future research needs to improve survival outcomes and preserve the quality of life for patients with BCBM.

## Introduction

Central nervous system (CNS) metastases are a devastating diagnosis for patients living with breast cancer. People living with breast cancer and CNS metastasis represent an understudied cohort of patients with unique challenges to manage their disease. CNS metastasis describes any metastases within the brain or the intramedullary spinal cord. This review will highlight the biology, current treatment strategies, and ongoing clinical trials for breast cancer that has metastasized specifically to the brain (BCBM). We will discuss unmet needs that leave patients living with BCBM feeling overlooked.

Breast cancer is the most common neoplasm among women and causes 500,000 deaths annually worldwide, with ~1.3 million new cases diagnosed each year^1^. Breast cancer is classified using pathological markers, TNM staging (tumor size, lymph node, and metastatic spread), and gene expression patterns^1^. Breast cancer is broadly classified by origin, either in the breast duct, in the case of intraductal carcinoma (IDC), or the breast lobule for intralobular carcinoma (ILC). Patients with ILC are reported to have a higher likelihood of bone, gastrointestinal and ovarian metastasis and less likely to have CNS, regional lymph nodes or lung metastasis as their first site of metastatic recurrence compared to patients with IDC.

In addition to characterization by origin, breast cancer has been molecularly characterized, initially by five main subtypes (Luminal A, Luminal B, Basal, HER2-enriched, Normal Breast-Like)^2,3^ which closely overlap with pathologically defined subtypes. Pathologists use immunohistochemical (IHC) staining to determine the presence or absence of two hormone receptors (HR), the progesterone receptor (PR) and the estrogen receptor (ER), as well as the human epidermal growth factor receptor 2 (HER2)^1,4^. IHC-defined tumor subtypes have been associated with a difference in a patients’ median survival at the time of a diagnosis of brain metastasis. Patients with HR+/HER2+, HR−/HER+, HR+/HER2−, and HR−/HER2− have a median survival of 21–27, 18–25, 10–14, and 6–9 months, respectively^5^. Molecular markers, like *BRCA1* or *BRCA2* (breast cancer gene 1 or 2) germline mutations, are indicators of possible basal-like breast cancer development and are used to determine potential risk and guide treatment^6^. Recent large-scale sequencing efforts have led to the identification of the genes with the highest mutation rates in breast cancer, including *TP53*, *PIK3CA*, *AKT1*, *PTEN*, *ERBB2*, *ATM*, *CDH1*, *APC*, *KRAS, NRAS*^7^. Ongoing research will determine whether these mutations are “actionable” in order to lay the foundation for personalized medicine approaches. The most common organ sites for breast cancer metastases are bone, brain, liver, and lungs^8,9^. Breast cancer is the second leading cause of all brain metastasis, and 10–16% of patients with advanced breast cancer have or develop brain metastasis^10^. National trials are underway to determine if early detection of brain metastasis would improve survival and quality of life, because current NCCN guidelines do not recommend brain imaging unless neurocognitive symptoms develop^11,12^. Patients are often not educated about the symptoms suggestive of cancer spread to the CNS. Failure to identify CNS metastasis early likely results in more invasive and toxic interventions such as whole-brain radiation therapy (WBRT) or surgical resection. There is increased support by the patient advocate and medical communities to include brain imaging at the time of a MBC diagnosis, especially in patients with an increased risk of developing BCBM. Koniali et al. provide an extensive review of the risk factors for BCBM^13^. The main risk factors include age (<49 years old), higher-grade cancer, prior visceral metastases, HER2-positive or triple-negative status, and mutations in the *BRCA1* gene.

The incidence of BCBM is rising^14^. Several contributing factors include increased detection due to improved and more widely available radiological techniques and targeted therapies to treat systemic disease, which prolong survival^14,15^. The extended survival time has led to a 25–40% increased incidence in brain metastasis in patients with HER2-positive breast cancer and as high as 46% among patients with advanced TNBC^14,16,17^.

BCBM most commonly occurs in the brain’s parenchyma (neurons and glia). The spread of cells to the pia mater, arachnoid mater, subarachnoid spaces, and the cerebral spinal fluid (CSF) is known as leptomeningeal metastasis or disease (LMD)^18^. LMD remains understudied because it occurs at a lower rate, is difficult to diagnose^19,20^, and is associated with a median survival of 15 weeks^20^. Le Rhun et al. compared 50 patients with LMD to 50 patients with breast cancer and no CNS metastases including LMD. The cohorts were matched based on their age at time of diagnosis, the year of diagnosis, and the type of chemotherapy that they received. Factors associated with risk for LMD included: lobular histology, HR-negative status, and metastasis at time of breast cancer diagnosis^21^. In a separate study, patients with TNBC as well as patients with higher grade cancer developed LMD within a shorter time frame as compared to patients’ with receptor positive tumors and/or lower grade tumors^22^. There are no standards for neurological examinations, neuro-imaging assessment, or a specific CSF cytological to diagnose LMD. A Response Assessment in Neuro-Oncology (RANO) working group has been established to develop these criteria. Finding the means to overcome CNS metastasis is, undoubtedly, an unmet clinical need for which more research is required.

## BASIC science research

### Animal models

Mouse models for breast cancer metastasis rely on the injection of human MBC cell lines or patient-derived organoids into immune-compromised mice. Several mouse cell lines derived from spontaneous mouse tumors are transplantable in a syngeneic murine background with a competent immune system^23^. Transgenic or knockout mice that develop spontaneous mammary carcinomas that metastasize have also been developed^24,25^. An alternative metastasis model was created using variants of human breast cancer cell lines serially injected via the heart or tail of mice and isolated from the bone, lung, or brain^26^. When tested in experimental metastasis models, by intracardiac injection, these variants showed preferential homing to the organ from which they were harvested^27^. BCBM experimental models can be established by injecting cells into the mouse brain or carotid artery^28,29^, and used to test the ability of therapies to treat CNS lesions effectively. The downside of such models is they circumvent the development of a primary tumor and bypass the initiating steps of metastasis^28^.

Clinically relevant models of LMD were established by Boire et al., who performed three serial rounds of the direct injection of human or mouse cancer cells into the cisterna magna and then collected the primed cells. The primed cells were intracardially injected into a separate cohort of animals, and the disseminated cells consistently formed LMD instead of CNS disease^30^. The group also discovered that cells in the CSF express complement component 3, promoting disruption of the brain-CSF barrier and predicting leptomeningeal relapse^31^. Kuruppu et al. established a model in which mice develop neurological symptoms that bear clinical resemblance to LMD. The model can be used to evaluate potential treatment strategies^32^. Preclinical models that replicate BCBM and LMD are improving, but the lack of spontaneous breast to brain models has stagnated research.

### Organotropism

Metastasis studies have focused on the concept of “organotropism”, or the ability for a cancer cell to preferentially home to, and survive in, a specific organ. Organotropism could occur due to circulation patterns, a pre-metastatic niche^33^, a symbiotic relationship of cancer cells with resident cells, a specific gene expression profile, or even the immune microenvironment of the organ^34,35^. Whether breast cancer cells have inherent organotropism initially in the primary tumor or whether they adapt to the metastatic site continues to be debated in the field^36^.

In a recent study, circulating tumor cells (CTCs) acquired from four patients with breast cancer were injected into the left cardiac ventricle of NSG mice^37^. The metastases that formed were isolated, dissociated, and underwent multiple in vivo intracardiac injections to select for cells primed to colonize either the lung, bone, or brain^37^. These CTC-derived brain metastatic cells had high expression of semaphorin (SEM4AD), which increased transmigration in a simulated in vitro blood-brain barrier (BBB) assay and an in vivo mouse model^37^.

In separate studies, to elucidate mechanisms of extravasation in the brain, nude mice were subjected to serial intracarotid injections of MDA-MB-231 and CN34 cell lines to establish cell lines primed for brain metastasis^38^. The metastatic brain cells had increased COX2 and EGFR ligand (heparin-binding EGF) expression that promoted BBB permeability via prostaglandin production^38^. Also, the breast cancer cell surface expressed a brain-specific sialyltransferase (ST6GALNAC5)^38^. The next step is to combine the results of these studies to develop an assay that can be used to assess the risk of BCBM development.

### Brain microenvironment

Using intravital imaging of intracranially injected MDA-MB-231 cells, Simon et al. observed microglia directly interacting with breast cancer cells, altering microglial morphology and disrupting normal brain electrophysiology^39^. Resident astrocytes and microglial cells express cytokines that promote breast cancer cell proliferation^40^. Conditioned media from rat neonatal and adult astrocytes enhanced cancer cell invasion in vitro due to secreted matrix metalloprotease-2 (MMP-2) and MMP-9, while inhibiting MMPs in the media decreased metastatic growth of breast cancer cells following intracardiac injection^41^. This suggests that secreted factors from astrocytes contribute to colonization within the CNS^41,42^.

Astrocyte-derived exosomes can promote chemokine production in breast cancer cells leading to enhanced proliferation and reduced apoptosis^43^. Cancer cells that adapt to the brain microenvironment by mimicking CNS cells have an increased chance of survival. For example, Neman et al. showed that some breast cancer cells express proteins typically expressed in neuronal cells and can even catabolize GABA into succinate to form NADH^44^. Furthermore, expression of reelin in HER2-positive cancer cell lines co-cultured with astrocytes had increased proliferation which was reversed with the knockdown of reelin and HER2^45^. In both an intracardiac and intracranial injection models, TNBC and HER2-positive breast cancer cells activated astrocytes by expressing truncated glioma-associated oncogene homolog one, which enhanced brain colonization and increased the expression of genes associated with stemness (CD44, Nanog, Sox2, Oct4)^46^.

Microglia/macrophages can directly interact with breast cancer cells that have metastasized to the CNS. For example, Andreou et al. intracerebrally injected 4T-1 cells in BALB/c mice and identified subsets of activated pro-inflammatory and anti-inflammatory microglia/macrophages. The group then selectively depleted the anti-inflammatory microglia/macrophage population using mannosylated clodronate liposomes, reducing brain lesions^47^. Another study exploring the interaction of BCBM cells and macrophages demonstrated that cathepsin S is a regulator of BCBM. Inhibiting cathepsin S in both cancer cells as well as macrophages significantly reduced BCBM^48^. An analysis of metastasis-associated macrophages in the brain parenchyma of mice revealed upregulated cytokine and Lymphotoxin β production that promoted M2 polarization of macrophage cells^49^. Thus, astrocyte, microglia, and macrophage interactions with breast cancer cells promote metastasis through altered neuroinflammatory responses in the brain^50^. Basic research has led to the identification of potential targets that could be used to develop therapies or to predict BCBM but to be tested clinically, patients with BCBM need to be included in clinical trials.

## Clinical and translational research

Patients with BCBM are often excluded from clinical trials because BCBM is linked with an increased mortality rate. Out of 1474 clinical trials for patients with breast cancer conducted from 1992 to 2016 in a review by Costa et al., only 29% included patients with CNS disease, and only 1% (16 studies) were designed to consider BCBM specifically^51^. In 2016, ASCO and Friends of Cancer Research (Friends) established a Brain Metastasis Working group to change the exclusionary nature of the current eligibility criteria. The group lobbied for the inclusion of patients with BCBM in trials suggesting that they could be stratified into three cohorts of patients during clinical trial design: (1) those with treated/stable BCBMs, (2) those who have active BCBMs, and (3) those who have LMD^52^. A fourth cohort consisting of patients that have not received prior treatment but have stable BCBM should be considered. While the ASCO-Friends guidance is welcomed by the patient advocate community, the majority of clinical trials restrict eligibility to those patients with stable BCBM whereas the majority of patients with BCBM have active disease and who are in dire need for a clinical trial. Retrospective and prospective exploratory analysis have been conducted within larger cohorts of patients enrolled in a clinical trial to identify the incidence rate, time to development, and overall survival time following a diagnosis of brain metastasis.

Several treatment approaches are available to patients with BCBM, including local and systemic therapies. The majority of patients diagnosed with BCBM will have one or more local treatments but will likely continue their systemic therapy or transition to a different systemic approach.

### Localized therapy

Localized treatments for BCBM include surgery, WBRT, and radiosurgery. WBRT is preferred when there are many metastatic lesions but does not come without risk. WBRT can cause neurocognitive complications (e.g., sensory deficits, headache, changes in mental status, cognitive disturbances, seizures, ataxia, and motor loss) and does not improve overall survival unless combined with surgery or chemotherapy^53^. Stereotactic radiosurgery (SRS) is a favorable alternative because of reduced cognitive impairments. Recent Phase III results presented at ASTRO 2020 demonstrated that SRS led to less cognitive decline than conventional WBRT even in patients with multiple lesions (more than 4) without compromising disease control. This serves as the foundation of an ongoing clinical trial comparing SRS with hippocampal-avoidant WBRT plus memantine for 5–15 BM (NCT03550391)^54^. The study includes patients with active brain metastases but excludes any patients with LMD, >15 BM on a volumetric T_1_ contrast MRI within the past 14-days or >10 metastasis on non-volumetric MRI. Emerging data suggest that SRS remains a reasonable alternative even for patients with a large number of BCBMs, with one series reporting utilization of SRS in a patient with as many as 34 brain metastases^55^. A clinical trial of patients with BCBM from non-small cell lung carcinoma, melanoma, and renal cell carcinoma demonstrated a benefit in overall survival from concurrent SRS with immune checkpoint inhibitors, suggesting more studies regarding the timing of SRS in BCBM could provide insight^56^. Surgery is reserved for patients who present with a limited number of large or symptomatic brain lesion(s). Surgery is typically followed by adjuvant radiation therapy (RT), either in the form of SRS or WBRT^57^. Following a randomized, controlled, phase III trial (*n* = 194 patients), post-operative SRS has become the standard of care due to reduced cognitive decline but similar survival benefit when compared to WBRT^58^. Another treatment option for metastatic brain lesions is laser interstitial thermal therapy (LITT), which remains highly experimental though the technology was established in 1990. It was not until the late 2000s that FDA approved two ablation systems used to treat primary or recurrent tumors and radiation-induced necrosis, which are only available at large medical institutions. LITT is used on deep-seated tumors but its use is limited by the high cost of the procedure^59,60^.

Current clinical studies are focused on improving the effectiveness of RT and assessing specific neurological impairments caused by RT. Radiosensitizers like motexafin gadolinium (produces reactive oxygen species), efaproxiral (induces low oxygen by binding to hemoglobin), and RRx-001, which dilates blood vessels and improves oxygenation to the tumor site, have demonstrated efficacy in the prevention of neurocognitive impairment^61^. Drugs like memantine, an Alzheimer’s prescription drug, have been utilized to block vascular damage and reduce side effects like dementia that result from WBRT treatment of the hippocampal region^62^. To assess the neurological impairments that result from radiation treatment, specific cognitive examinations that establish a baseline and evaluate changes should be developed^63^.

Since patients with HER2-positive disease and TNBC have a higher rate of BCBM, prophylactic cranial irradiation (PCI), which has historically been utilized for patients with small-cell lung cancer and brain metastasis, is now being considered^14,64^. Some clinicians are concerned that PCI may be associated with too many adverse effects, including a risk to the patient’s quality of life^65^. Thus, the application of PCI remains controversial in the field, and clinical trials would be warranted. A partial list of active clinical trials that include a RT component are highlighted in Table 1.

**Table 1:** A partial list of ongoing clinical trials that include radiation therapy for patients with active and/or stable disease.

Trial #	Trial name	Recruiting (Y/N)	LMD eligible (Y/N)
NCT03190967	T-DM1 alone versus T-DM1 and metronomic temozolomide in secondary prevention of HER2-positive breast cancer brain metastases following stereotactic radiosurgery	Y	N
NCT03483012	Atezolizumab + stereotactic radiosurgery in triple-negative breast cancer and brain metastasis (specific to patients with active disease)	N	N
NCT03550391	Stereotactic radiosurgery compared with hippocampal-avoidant WBRT plus memantine for 5–15 brain metastases	Y	N
NCT04192981	GDC-0084 with radiation therapy for people with PIK3CA-mutated solid tumor brain metastases or leptomeningeal metastases	Y	Y
NCT04588246	Testing the addition of whole brain radiotherapy using a technique that avoids the hippocampus to stereotactic radiosurgery in people with cancer that has spread to the brain and come back in other areas of the brain after earlier stereotactic radiosurgery	Y	N
NCT04895592	Radiosurgery before surgery for the treatment of brain metastases	N	N
NCT04899908	Stereotactic brain-directed radiation with or without aguix gadolinium-based nanoparticles in brain metastases	N	N
NCT04923542	Stereotactic radiation & abemaciclib in the management of HR+/HER2− breast cancer brain metastases	N	N

### Targeted therapies

The systemic treatments often used to manage BCBM include corticosteroids to reduce cerebral edema and standard chemotherapy agents such as capecitabine, carboplatin, gemcitabine, and methotrexate^66^. Doxorubicin, cyclophosphamide, fluorouracil, paclitaxel, docetaxel, and vinorelbine may also be used but have poor blood-brain barrier penetrance^11^.

Systemic treatments for patients with BCBM beyond chemotherapy now include a small but quickly growing arsenal of targeted therapies. For example, small molecule inhibitors are being used to treat patients with HER2-positive cancer, and endocrine therapy combined with the cyclin-dependent kinase (CDK)4/6 inhibitor, abemaciclib, for patients with hormone receptor-positive disease.

A retrospective analysis of EMILIA trial data showed that the rate of CNS progression was similar (extracranial ORR), but the median overall survival was significantly improved in patients with asymptomatic CNS metastasis that were treated with trastuzumab emtansine (T-DM1) compared to lapatinib plus capecitabine^67^. Likewise, in the CLEOPATRA trial, an exploratory analysis demonstrated no difference in incidence, but the time to develop CNS metastases was prolonged from 11.9 to 15 months when pertuzumab was added to a trastuzumab and docetaxel treatment regimen^68^. The NALA trial compared the progression-free survival (PFS) of 101 patients with stable BCBM treated with either neratinib (*N* = 51) or lapatinib (*N* = 50) in combination with capecitabine. Patients with BCBM treated with neratinib had a median PFS of 7.8 months compared to 5.5 months for patients treated with lapatinib^69^. Results from the HER2CLIMB trial show that the median PFS in patients with active BCBM at baseline that received tucatinib, trastuzumab, and capecitabine was 7.6 months compared to 5.4 months in patients who did not receive tucatinib^70^. In a subgroup analysis of the DESTINY-Breast01 trial, a phase 1 dose-finding study for trastuzumab deruxtecan (T-DXd), 24 of the 184 patients enrolled had stable CNS metastasis. The objective response rate was 58.3%, and median PFS was 18.1 months for these patients^71^. T-DM1, T-DXd, neratinib, and tucatinib are FDA-approved treatments for patients with HER2-positive MBC that has progressed on prior HER2-targeted therapy(ies).

At the time of publication, globally, 230 ongoing clinical trials include patients with BCBM of which 36 are open to patients with LMD. The majority of the studies include targeted therapy such as antibody drug conjugates (ADCs), immunotherapies, novel chemotherapeutics, and small molecule inhibitors (Table 2). Table 3 highlights a partial list of recently reported BCBM clinical trial results. Table 4 highlights a partial list of active trials at the time of publication. For an up-to-date list of recruiting and active trials, please see both the patient-managed clinical trial database at TheStormRiders.org and the US-based Metastatic Breast Cancer Trial Search at BreastCancerTrials.org.

**Table 2:** Standard and emerging alternatives to current chemotherapies for patients with BCBM: Tyrosine kinase inhibitors^92–95^, CDKi^96^, PI3Ki^97^, PARPi^98–101^, novel chemotherapeutics^102–104^, and ADCs^105–107^.

Classification	Small molecule inhibitors				Novel chemotherapies	Antibody-drug conjugates (ADCs)
Target	TKs	CDK	PI3Ks	PARP inhibitors	Inducing cytoxicity	Selectively binds to tumor via receptor/or marker
Drug names	Asciminib/ABL001, epertinib, pyrotinib, E01001	Abemaciclib, Palbociclib	GDC-0084	Iniparib, niraparib, olaparib, veliparib	Nal-IRI/MM398, NKTR-102, Temozolomide, Tesetaxel	T-DM1 (Kadcyla), IMMU132 (Trodelvy), DS-8201 (Enhertu)

**Table 3:** A subset of clinical trials that included patients with BCBM field.

Trial #	Design	Drug or Therapy	Result	Conclusion
NCT01494662 Freedman et al. 2019^108^ Phase II	HER2+ BCBM: Cohort 3A lapatinib-naïve (*N* = 37 active CNS disease, *N* = 2 LMD); Cohort 3B were lapatinib-treated (*N* = 12 active CNS disease, *N* = 1 LMD). *N* = 168 (overall)	Neratinib (240 mg/day) + capecitabine (750 mg/m^2^ bid)	CNS ORR Cohort 3A = 49% (*N* = 8 active CNS); Cohort 3B = 33% (*N* = 5 active CNS)	Neratinib plus capecitabine is active in refractory, HER2+ BCBM
NCT01808573 (NALA) Saura et al.^109^ Phase III	Refractory HER2+ MBC: neratinib + capecitabine (*N* = 51 stable BCBM) vs. lapatinib + capecitabine (*N* = 50 stable BCBM) *N* = 621 (overall), open label	Neratinib (240 mg/day) + capecitabine (750 mg/m^2^ bid) vs. lapatinib (1250 mg/day) + capecitabine (1000 mg/m^2^ bid)	Neratinib vs. lapatinib, extracranial ORR was 33% vs. 27% respectively; Rates of PFS were 47% vs. 38% at 6 mo; 29% vs. 15% at 12 mo; and 16% vs. 7% at 18 mo	The neratinib/capecitabine combination reduced the risk of disease progression or death by 24%
NCT02025192 Murthy et al.^70^ Phase I	HER2+ stable MBC: tucatinib (300 mg) plus capecitabine (*N* = 2 stable BCBM), tucatinib (300 mg) plus trastuzumab (*N* = 16 stable BCBM), tucatinib (300 mg) plus capecitabine and trastuzumab (*N* = 11 stable BCBM), tucatinib (350 mg) plus capecitabine (*N* = 3 with BCBM), tucatinib (350 mg) plus trastuzumab (*N* = 1 with BCBM) *N* = 60 (overall)	Capecitabine (1000 mg/m^2^/day), Trastuzumab (2 mg/kg IV), Tucatinib (2×/day 300 or 250 mg)	Tucatinib MTD 300 mg 2×/day. Extracranial ORR: comb-1 83%, comb-2 40%, comb-3 61%	Acceptable toxicity, antitumor activity. Led to double-blinded randomized study, HER2CLIMB (NCT02614794) Drug combination was FDA approved on April 17, 2020 for adult pts with HER2+ MBC
NCT02308020 Anders et al.^110^ and Tolaney et al. 2020^111^ Phase II	BC, NSCLC, or melanoma w/active BM. Cohort A: HR+/HER2− (*N* = 58 active BCBM), Cohort B: HR+/HER2+ (*N* = 27 active BCBM), Cohort C: HR+/HER2− (*N* = 7 active LMD) and HR+/HER2+ (*N* = 3 active LMD) *N* = 162 (overall)	Abemaciclib (200 mg every 12 h/21 days)	Out of 52 evaluable pts, 3 pts (6%) had objective intracranial RR, 25% Intracranial clinical benefit rate	Abemaciclib has intracranial clinical benefit in heavily pretreated HR+, HER2− MBC pts w/BM. (Note: HER2+ cohort did not pass 1st stage)
NCT02312622 Zimmerman et al.^112^ Phase II	Refractory BM and advanced lung cancer/active MBC. NSCLC, SCLC, and MBC (*N* = 12 overall and *N* = 2 with active BCBM). *N* = 27 (overall)	Pegylated Irinotecan (IV 145 mg/m^2^/21 days)	CNS disease control rate as well as overall disease control rate two pts (16.7%) NCSLC and two pts MBC (16.7%)	A low overall disease control and CNS disease control rate. May help a small subset of pts.
NCT02536339 Lin et al.^113^ Phase II	HER2+ MBC with active CNS progression post-radiotherapy. Single group assignment. *N* = 40 (overall and with active CNS disease, LMD excluded)	Pertuzumab (840 mg loading dose + 420 mg by IV every 3 week), Trastuzumab (IV 6 mg/kg)	Four pts confirmed partial response, intracranial ORR of 11%.	The CNS ORR of 11%. May have clinical utility
NCT02650752 Morikawa et al.^114^ Phase I	HER2+ active BCBM and/or LMD. Single group assignment: parenchymal BM (*N* = 4), LMD (*N* = 5), and spinal cord (*N* = 2) *N* = 11 (overall), open label	Lapatinib (increasing dose days 1–3 and days 15–17) + capecitabine (days 8–14 and day 22-2 at 1500 mg)	Four pts BM only, 5 pts LMD, 2 pts intramedullary spinal cord. MTD lapatinib 1500 mg	High-dose lapatinib is tolerable with capecitabine. Antitumor activity in intracranial CNS as well as non-CNS sites of disease
NCT2614794 (HER2CLIMB) Murthy et al.^70^ Phase II	Refractory HER2+ active MBC: Tucatinib (*N* = 20 with active BCBM)or placebo + capecitabine + trastuzumab (*N* = 55 with active BCBM) *N* = 612 (overall), open label	Tucatinib (2×/day 300 mg), capecitabine (1000 mg/m^2^/day), trastuzumab (8 mg/kg IV),	ORR was significantly higher in the tucatinib arm vs. the control arm (41% vs. 23%); Median OS was 21.9 mo vs. 17.4 mo; Median PFS was 7.6 mo vs. 5.4 mo	Addition of tucatinib to trastuzumab + capecitabine in heavily pretreated pts with HER2+ MBC, including those with BCBM, was associated with statistically significant PFS & OS
NCT01921335 Metzger Filho et al.^115^ Phase I	HER2+ active BCBM and HER2+ MBC: Tucatinib + trastuzumab *N* = 41 (overall and with active CNS disease, LMD *N* = 2 l),open label, randomization	Tucatinib (21-day), trastuzumab (6 mg/kg q3w; after an 8 mg/kg loading dose if >5 wks since last trastuzumab)	CNS intracranial ORR ~6%, median PFS was 2.4 months, 14.7% pts progression free at 4 mo	Combination of tucatinib with trastuzumab is tolerable and showed efficacy in pts with HER+ BCBM

*HR* hormone receptor, *MBC* metastatic breast cancer, *MTD* maximum tolerated dose, *ORR* objective response rate, *PFS* progression free survival, *NSCLC* non-small cell lung cancer, *OIRR* overall intracranial response rate, *S* overall survival, *SCLC* small cell lung cancer, *pts* patients, *mo* months, *h* hours, *wk* weeks.

**Table 4:** A partial list of active clinical trials investigating therapeutic options for breast cancer patients with BCBM or LMD.

Subtype	Trial #	Treatment	Trial name	Recruiting (Y/N)	BCBM status for eligibility	LMD eligible (Y/N)
HER2+	NCT03054363	HER2i, HER2i	Tucatinib, palbociclib, and letrozole in Metastatic HR+ and HER2+ breast cancer	N	Stable	N
	NCT03417544	IT, CT, HER2ab	Atezolizumab + Pertuzumab + Trastuzumab in CNS Mets	N	Both	N
	NCT03501979	HER2i, HER2ab, CT	Tucatinib, Trastuzumab, and Capecitabine for the treatment of HER2+ LMD	N	Active (LMD)	Y
	NCT03765983	HER2ab, PI3Ki	GDC-0084 in combination with trastuzumab for patients with HER2+ BCBM	Y	Both	N
	NCT03933982	RTKi, CT	A study of pyrotinib plus vinorelbine in patients with brain metastases from HER2+ metastatic breast cancer	Y	Stable	N
	NCT03975647	HER2i, HER2ab	A Study of Tucatinib vs. Placebo in Combination with Trastuzumab Emtansine (T-DM1) for patients with advanced or metastatic HER2+ breast cancer	Y	Both	N
	NCT03696030	IT	HER2-CAR T cells in treating participants withbrain or leptomeningeal metastases	Y	Both	Y
	NCT04420598	ADC	DS-8201a for treatment of ABC, brain metastasis, and HER2+ disease	Y	Both	Y
	NCT04704661	HER2ab, STKRi	Testing the combination of DS-8201a and AZD6738, for the treatment of patients with advanced solid tumors expressing the HER2 protein or gene	Y	Stable	Y
	NCT04721977	HER2i, HER2ab, CT	A study of Tucatinib (MK-7119) in combination with Trastuzumab and capecitabine in participants with previously treated locally advanced unresectable or metastatic HER2+ BC (MK-7119-001)	Y	Stable	N
	NCT04739761	ADC	A study of T-DXd in participants with or without BM who have been previously treated, advanced or metastatic HER2+ BC	Y	Stable	N
HER2−	NCT03613181	PDC	ANG1005 in leptomeningeal disease from BC	N	Stable	Y
ALL	NCT03994796	–	Genetic testing in guiding treatment for patients with BM	Y	Stable	N
ALL	NCT03995706	ADC	Neuro/Sacituzumab Govitecan/BCBM/Glioblastoma	Y	Active	N
ALL	NCT04396717	Antibody	Safety study of pritumumab in brain cancer	Y	Active	Y

*HER2i* HER2 inhibitor, *IT* immunotherapy, *CT* chemotherapy, *HER2ab* HER2 antibody, *HER2ab* HER2 inhibitor, *PI3Ki* PI3K inhibitor, *RTKi* receptor tyrosine kinase inhibitor, *ADC* antibody-drug conjugate, *STKRi* serine/threonine kinase receptor inhibitor, *PDC* peptide-drug conjugate, *BC* breast cancer, *BM* brain metastasis, *HR* hormone receptor.

### Immunotherapy

There is a broadening interest in using immune checkpoint inhibitors as therapeutics for BCBM after preliminary studies showed efficacy in other solid tumor types^72^. A retrospective study of 84 BCBM biopsies demonstrated that PD-L1 and PD-L2 were expressed in 53 and 36% patients, respectively, suggesting immune checkpoint inhibitors could provide a therapeutic benefit^73^. Another viable immunotherapy uses chimeric antigen receptor-engineered T (CAR T) cells that have been optimized in a xenograft mouse model of BCBM. The HER2-CAR T cells reduced T-cell exhaustion in vivo and intracranial delivery demonstrated antitumor efficacy^74^. This research has led to an ongoing clinical trial (NCT03696030) for patients with active brain or leptomeningeal metastases. Other immunotherapies include vaccines that introduce neoantigens that are specific to glioma. When viable neoantigens are identified for BCBM, the same strategy could be employed^75^.

### Therapeutics that cross the blood-brain barrier (BBB)

One main obstacle for identifying efficacious BCBM therapies is the BBB. BCBM development occurs when cancer cells detach from the primary tumor, invade, cross the endothelial barrier, survive in the bloodstream, extravasate, and grow at the secondary site^76^. The capillaries that make up the BBB are different from the endothelium in other organs. The BBB is composed of endothelial cells with tight junctions, no fenestrations or pinocytic vesicles, and are encased in a basal membrane and extracellular matrix barrier^77^. Apart from size, polarity (nonpolar preference) and lipophilicity contribute to the restrictions for passive diffusion^77^. We refer the readers to a review by Deeken and Loscher that discusses ways to overcome the BBB using transporter inhibition, nanoparticles, immunoliposomes, peptide vectors, or carrier-mediated active transport mechanisms^77^. We also note a retrospective review of animal and human studies of HER2-positive BCBMs by Kabraji et al. that revealed a drug’s ability to cross the BBB did not necessarily correlate with efficacy^78^, suggesting additional factors contribute to the inadequate response.

### Local administration of drugs

The direct injection of therapeutics into the spinal canal or subarachnoid space has improved drug efficacy in some instances^79^. For example, a case study reported on a patient with HER2-positive breast cancer and multiple brain lesions treated with intrathecally-delivered trastuzumab, resulting in stabilizing brain and epidural metastases^80^. A phase 1 clinical trial (NCT01325207) was initiated to test intrathecal delivery of trastuzumab and identify a maximum tolerated dose for patients with LMD and stable systemic disease in HER2+ breast cancer^81^. Another method of direct CNS delivery is convection-enhanced delivery (CED) via a pressure gradient at the tip of an infusion catheter. Still, this method has not been largely successful due to the dependence on volume and rate of gradient infusion, resulting in an uneven distribution of drugs or potential drug efflux from the injection site and toxicity from treatment^82^. One final strategy for increasing CNS drug delivery is ultrasound-induced BBB opening that has shown some preclinical efficacy as well as efficacy for CNS diseases such as gliomas^82^. Intrathecal delivery is largely limited to patients with HER2+ disease as other agents (e.g., chemotherapy) can cause debilitating toxic side effects with limited benefit and are unsustainable for indefinite use.

### Imaging

MRI is most commonly used to monitor disease progression and side effects from treatment, detect recurrence, and identify new metastases post-treatment. Still, current imaging techniques lack the power to differentiate pseudo-progression from actual progression. Improving imaging techniques to diagnose and monitor BCBM earlier could enhance the quality of life for patients^11^. A recent study using AMT-PET imaging successfully differentiated primary brain tumors and metastatic brain tumors with greater than 90% accuracy launching a clinical study for patients with BCBM to improve diagnoses by enhancing the ability to differentiate abnormal and normal tissue (NCT01302821)^83^. Artificial intelligence (AI) will likely play a future role in assessing treatment response of brain tumors; Machine learning methods carefully trained on standard MRI could be more reliable and precise than established methods^84^.

### Liquid biopsies

To identify patients most at risk to develop BCBM, researchers are advancing the capabilities of liquid biopsies since actual BCBM biopsies are an impossibility in most cases^85^. Riebensahm et al. showed a significant association of decreased overall survival when CTCs were detectable^86^. Cell-free DNA (cfDNA) has been used to assess genomic alterations. To identify genetic mutations, cfDNA was isolated and sequenced in blood samples from 13 patients with BCBM and 36 patients without BCBM^87^. There was a high correlation of mutations in *APC, BRCA1*, and *CDKN2A* associated with BCBM, which provided supportive evidence for cfDNA biomarkers^88^. In a study of 194 patients with MBC cfDNA and CTCs were compared for their ability to predict PFS, OS, and response to treatment. cfDNA was simpler to isolate, more informative, and less expensive than isolating and quantifying the number of CTCs^89^. Proteins in the serum or CSF of patients have also been considered as predictive biomarkers of metastasis. For example, Dao et al. identified increased (>0.1 ng/mL) levels of the angiopoietin-like fibrinogen-like domain (cANGPTL4) in the serum of patients with breast cancer, which was associated with increased risk for BCBM^90^.

## Patient perspectives: care gaps and future research needs

### Patients living with central nervous system (CNS) metastasis

Due to a lack of guidelines for the treatment of CNS metastasis, once a diagnosis is confirmed, treatment is at the discretion of a patient’s oncology team. Most cancer patients are treated locally in community hospitals. They are not likely to have access to a breast oncologist specializing in CNS metastasis or a multidisciplinary team who form a consensus on treatment decisions. Even with access to a multidisciplinary team, which often includes a medical oncologist, neuro-radiation oncologist, neuro-oncologist, and neurosurgeon, the continuity of care is often a problem for patients because the burden of facilitating communication between the three specialties falls on the patient themselves. Treatment options are limited to invasive interventions that can cause debilitating side effects and seriously impact the quality of life. Despite having access to the “best” care, patients with CNS metastasis still have a worse prognosis, disproportionate treatment response, and lower overall survival than patients with metastasis in other organs. The disparities in the quality of care are even more significant among patients with low socioeconomic status as well as patients identified as racial/ethnic minorities—African Americans, American Indians, and Alaskan Natives, Asians, Native Hawaiians/other Pacific Islanders, and Hispanics/Latinos^91^.

Patients living with CNS metastasis represent a vulnerable cohort, and when medicine and research fail this understudied community, they often turn to other patients and advocates for solutions. Therefore, patient advocates have been charged with leading the effort to address the care gaps and research needs of breast cancer patients who develop CNS metastasis. Though many assume patient advocacy is synonymous with support (e.g., emotional, financial, and educational) patients have become increasingly valuable to the medical and research community, because they offer a unique perspective as experts living with cancer. As the ultimate end users of products developed through research, patients can, and have, helped drive more impactful research that improves survival outcomes.

### Future research needs

In 2020, the MBC Alliance patient advocates recognized an opportunity to capitalize on the momentum gained from the approval of tucatinib (Tukysa^®^), the first and only drug approved for BCBM. The Alliance launched the patient-led BCBM Initiative: Marina Kaplan Project, in memory of Marina Pomare Kaplan, with the overarching goal to identify the unmet research needs of patients living with CNS metastasis. The project includes members with representation from industry, research organizations, and individual patients. Nearly one-third of the group is comprised of patients living with brain metastases or LMD. The 17-member scientific advisory board, comprised of a multidisciplinary array of experts in the field of brain metastasis and LMD from breast cancer, advised on the identification of the following gaps in CNS-metastasis research: 1) a poor understanding of the unique brain microenvironment, 2) the absence of sufficient preclinical in vivo animal models that mimic multiple aspects of brain metastasis in a clinical setting, 3) the inability of many anticancer agents to cross either the blood-brain or blood-tumor barrier, 4) the lack of clinically meaningful endpoints that measure survival and quality of life, and 5) the lack of representation of patients with brain metastasis in clinical trials due to restrictive eligibility criteria. Future work addressing each of these gaps will be essential to reduce deaths due to brain metastasis.

## Concluding remarks

Over the past 20 years, basic research in breast cancer metastasis has led to identifying candidate genes whose expression is predictive of metastasis to specific organs. Prospective studies are warranted to develop assays that detect biomarkers linked to metastatic outcomes. Such improvements would be invaluable for decisions regarding clinical treatment and monitoring.

Advances in clinical trial design have allowed subgroup analyses to determine the effectiveness of new therapies of smaller patient subpopulations over the last decade. These analyses have led to the FDA approval of several HER2-targeted treatments that have efficacy for patients with BCBM. Still, much work is needed, particularly to extend outcomes beyond months into years and to consider TNBC. An integrated approach to cancer research that includes the voice of patient advocates will allow us to tackle the remaining challenges while improving the lives and outcomes for patients with breast cancer.
